# A Randomised Study To Compare Palonosetron With Ondansetron for Prophylaxis of Postoperative Nausea and Vomiting (PONV) Following Laparoscopic Gynecological Surgeries

**DOI:** 10.7759/cureus.23615

**Published:** 2022-03-29

**Authors:** Rohit Balyan, Sachin Kumar, K Lalitha, Sanjeev Aneja, Jai George

**Affiliations:** 1 Anesthesiology, Maulana Azad Medical College, New Delhi, IND; 2 Anesthesiology, All India Institute of Medical Sciences, New Delhi, New Delhi, IND; 3 Anesthesiology, Indraprastha Apollo Hospitals, New Delhi, IND

**Keywords:** general anesthesia, gynecological surgery, laparoscopy, postoperative nausea and vomiting, ondansetron, palonosetron

## Abstract

Background

Incidence of postoperative nausea and vomiting (PONV) in susceptible patients can be unacceptably high (70-80% reported incidence). This study was designed to evaluate the effect of palonosetron and ondansetron in preventing PONV in high-risk patients undergoing gynecological laparoscopic surgery.

Methodology

In this randomized, controlled, double-blind trial, non-smoking females aged 18-70 years, weighing 40-90 kg, and posted for elective laparoscopic gynecological surgeries were enrolled into ondansetron (Group A, n = 65) and palonosetron (Group B, n = 65) groups. Palonosetron (1 mcg/kg IV) or ondansetron (0.1 mg/kg IV) were administered just before induction. Postoperatively, the incidence of nausea, vomiting, PONV (scored on a scale of 0-3), need for rescue antiemetic, complete response, patient satisfaction, and adverse effects were evaluated up to 48 h following surgery. Normally distributed continuous variables were compared using Student’s t-test. In addition, the Chi-squared test or Fisher’s exact test were used to compare nominal categorical data as deemed appropriate. P-value <0.05 was observed as statistically significant.

Results

The overall PONV scores and postoperative nausea scores during 0-2 and 24-48 hours were comparable, but PONV scores (p = 0.023) and postoperative nausea scores (p = 0.010) during 2-24 hours were significantly lesser in Group B compared to Group A. There was no statistically significant difference in the postoperative vomiting score or retching during 0-48 hours. The amount of first-line rescue antiemetic used during 2-24 hours was significantly higher in Group A (56%) than in Group B (31%) (p = 0.012; p <0.05). A complete response to the drug during 2-24 hours was significantly higher (p = 0.023) in Group B (63%) compared to Group A (40%), whereas response was comparable during 0-2 and 24-48 hours. Both groups had a comparable incidence of adverse effects and patient satisfaction scores.

Conclusion

Palonosetron has a superior anti-nausea effect, less need for rescue antiemetics, and lesser incidence of total PONV compared to ondansetron during 2-24h and comparable effect to ondansetron during 0-2h and 24-48h postoperative period in high-risk patients undergoing gynecological laparoscopic surgery.

## Introduction

Anesthesia practice has improved significantly in the last few decades owing to the advancement in drug therapy. However, postoperative nausea and vomiting (PONV) remain a distressing symptom second only to pain [1]. PONV can lengthen hospital stay and cause delayed recovery. In addition, in cases with prolonged vomiting, morbidities including pulmonary aspiration, bleeding, wound dehiscence, and dehydration can lead to adverse consequences.

There is multifactorial etiology and pathophysiology of PONV that involves multiple receptor pathways. Risk factors identified in Apfel's simplified risk scoring system increase the likelihood of PONV by 18-22% per risk factor, emphasizing the significance of prevention and control by anesthetists [2]. Laparoscopic surgeries are now emerging as the preferred technique for diagnostic and/or therapeutic gynecological procedures. However, the incidence of PONV is high with such procedures (40-75%) [3].

Traditional antiemetics like phenothiazines, antihistamines, metoclopramide, and droperidol have been replaced by newer 5-Hydroxytryptamine type-3 receptor antagonists (5-HT3RA) owing to their higher efficacy, longer/sustained activity, and favorable side effect profile [4,5]. Among these, ondansetron is the most frequently used drug. Recently, second-generation 5-HT3RA palonosetron has been reported to have better receptor binding affinity and a very long plasma half-life of 40 hours, allowing extension of anti PONV effect to second and third postoperative days [6-8].

Although recent literature supports the use of either ondansetron or palonosetron, certain studies support the use of one over the other. For patients with high-risk factors, antiemetic efficacy and potency of palonosetron prophylaxis remain debatable in the late postoperative period [9,10]. Also, some studies comparing the effectiveness of palonosetron with ondansetron in PONV prophylaxis following laparoscopic surgery have shown controversial results and need further research to provide better clinical evidence [8,11]. So, we undertook this study to gather more data to evaluate and compare the efficacy of palonosetron with ondansetron for PONV prophylaxis for 48 hours in high-risk patients undergoing laparoscopic gynecological surgery.

## Materials and methods

**Figure 1:**
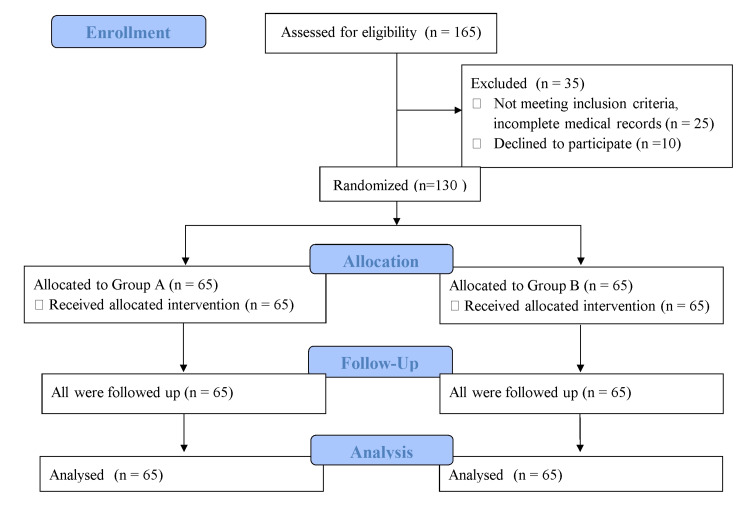
Consort flow diagram.

We conducted this study over one year after obtaining approval from the hospital's Institutional Ethics Committee, Clinical trial registration number (CTRI/2018/03/012655). Female patients aged 18-70 years, belonging to the American Society of Anesthesiologists (ASA) grade I-II planned for laparoscopic gynecological surgeries, non-smoker, and weighing 40-90 kgs were enrolled in the study. Written informed consent was obtained. Exclusion criteria included weight >90 kg, history of PONV, motion sickness, known hypersensitivity to study drugs, evidence of major organ dysfunction, pregnancy, lactation, and existing GI disease. In addition, patients who were already on antiemetics, steroids, or psychomimetic drugs preoperatively, on chemotherapeutic agents in the last few weeks, and those who were unable to cooperate and unwilling to participate in the study were also excluded (Figure 1).

This is a prospective, randomized, controlled, double-blind study. We used simple randomization (chit-in-box system) to divide the patients into two groups of 65 each. Group A received ondansetron 0.1 mg/kg (maximum 8 mg intravenously IV) and group B received palonosetron 1 mcg/kg (maximum 75 mcg IV) [12]. The anesthesiologist/investigator and the patients involved in the study were blinded to group allotment. The syringes (labeled "antiemetic," diluted with normal saline, the total volume of 5 ml of clear fluid) containing the study drug were prepared by an anesthesiologist not involved in the study. All patients had more than three risk factors (female, non-smoker, postoperative opioid use, and laparoscopic gynecological surgery under general anesthesia) and hence, came under the high-risk category for PONV [2,13].

All the patients were allowed to take a light and nonresidual diet on the evening of the previous day, and clear liquids were given 2 to 4 hours before the surgery. The preanesthetic regimen consisted of fasting as per ASA task force guidelines for preoperative fasting, and IV balanced salt solution (Ringer's lactate solution) was started in the preoperative room 20 minutes before the scheduled time of surgery (considering fasting duration and maintenance requirements based on body weight). Anesthesia procedure was standardized for all. After recording baseline vitals, patients received the respective antiemetic drugs just before induction. Induction of anesthesia was achieved with fentanyl 2 mcg/kg IV and propofol (1%) 1.5-2 mg/kg IV, and endotracheal intubation was facilitated by atracurium (0.5 mg/kg) IV. Intraoperative monitoring involved electrocardiography, non-invasive blood pressure, pulse oximetry, and capnography (EtCO2). The intraoperative fluid therapy with isotonic balanced crystalloid solution (Ringer's lactate solution) was standardized for all patients as per the 4-2-1 rule for perioperative fluid therapy (liberal fluid management strategy), and replacement of any blood loss and third space losses was done. Anesthesia was maintained with controlled mechanical ventilation (EtCO2 between 30 and 40 mmHg) and anesthetic gases (sevoflurane in 50% oxygen and air). At the completion of the surgery, residual neuromuscular blockade was reversed with neostigmine 0.05 mg/kg IV and glycopyrrolate 0.01 mg/kg IV, and the trachea was extubated. Multimodal analgesia was instituted with morphine 1 mg IV (SOS basis in a post-anesthesia care unit [PACU]), paracetamol 1 gm IV six-hourly, and diclofenac 1 mg/kg IV eight hourly in the ward.

An episode of PONV was defined as either a spell of nausea (unpleasant sensation with an urge to vomit), retching (involuntary, labored, spasmodic contractions of the respiratory muscles without expulsion of stomach contents), or vomiting (forceful expulsion of stomach contents from the mouth), and scored on a scale of 0-3 as per scoring system (Table 1) [14,15].

**Table 1:** The scoring system used for assessing postoperative nausea, vomiting, and PONV. PONV: Postoperative nausea and vomiting.

Score	Postoperative Nausea score	Postoperative Vomiting score	PONV score
0	None	None	No nausea/vomiting/retching/no rescue antiemetic required
1	Mild, intermittent nausea	One vomit only	Nausea
2	Constant, moderate nausea	Several vomits	Retching
3	Severe nausea	Repeated retching/vomiting	Vomiting

All data were collected for 0-2 hours in PACU and from 2 to 48 hours (2-24 and 24-48 hours) in the postoperative ward. Complete response was specified as there was no need to administer rescue antiemetics due to the absence of PONV. "Treatment failure" implied patients who experienced PONV despite receiving antiemetics. First-line rescue antiemetic drug in both groups (ondansetron 4 mg IV) was given for PONV and repeated after 30 minutes if symptoms persisted, followed by second-line or ultimate rescue antiemetic drug (dexamethasone 4 mg IV). Ondansetron was used as a first-line rescue antiemetic due to the slow onset of action of dexamethasone. Drug-related adverse effects (headache, dizziness, drowsiness, constipation, and ECG changes) were recorded. Rating for overall satisfaction after surgery (satisfied, neutral, dissatisfied) was enquired from the patients.

The primary outcome measured in our study was the incidence of overall PONV, postoperative nausea, and vomiting in the first 48 hours following surgery. Secondary outcomes were the requirement of rescue antiemetics (total amount administered), complete response to study drugs, patient satisfaction score, and incidence of adverse effects.

We calculated the sample size based on the observed incidence of PONV during 24 hours. Using an alpha value (0.05) and power of 80%, 65 patients per study group were found to be sufficient to detect a significant difference of 25% in the incidence of PONV between palonosetron and ondansetron groups [12,16]. We performed statistical testing with SPSS (Version 17.0. SPSS Inc., Chicago, US). Continuous variables were expressed as mean ± SD and categorical variables as absolute numbers and percentages. Normally distributed continuous variables were compared using Student's t-test. Chi-squared test or Fisher's exact test were used to compare nominal categorical data as deemed appropriate. P-value <0.05 was observed as statistically significant.

## Results

The study enrolled 130 patients with no dropouts. There were no statistically significant differences between the study groups in patient characteristics and anesthesia time. Preoperative, intraoperative, and postoperative vitals recorded were comparable between the study groups. There was no difference in postoperative morphine requirement between both the groups, and none of the patients received more than one dose of morphine (Table 2).

**Table 2:** Patient characteristics, duration of anesthesia, and morphine requirement in PACU. Data are mean ± SD or numbers of patients (%). ASA grade: American society of anesthesiologists grade; PACU: Postanesthesia care unit.

	Group A (n = 65)	Group B (n = 65)	P-value
Age (years)	37.40 ± 9.59	39.51 ± 8.67	0.191
Weight (kg)	64.90 ± 11.10	65.10 ± 8.53	0.909
ASA grade (I/II)	32/33 (49.2%/50.8%)	39/26 (60%/40%)	0.218
Duration of anesthesia (minutes)	150.85 ± 57.42	145.08 ± 36.23	0.495
Morphine requirement in PACU	20 (30.76%)	16 (24.61%)	0.435

**Figure 2:**
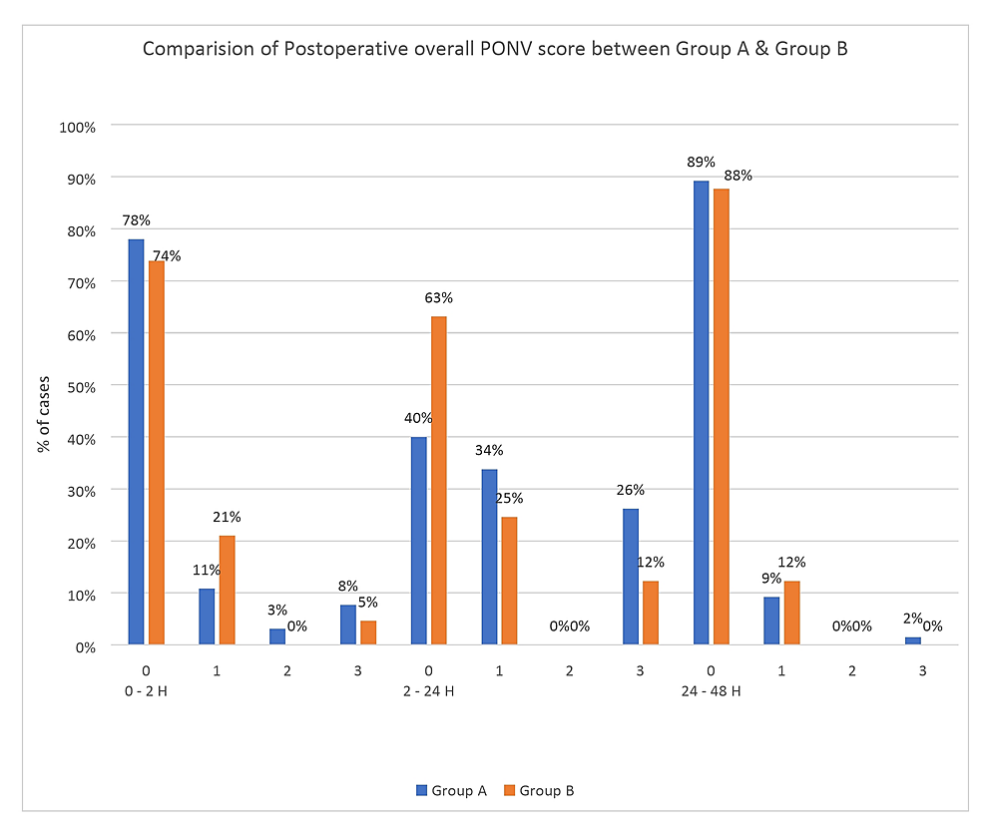
Comparison of postoperative overall PONV score between Group A (ondansetron) and Group B (palonosetron). 0,1,2,3 on x-axis denotes PONV scoring system used at various time intervals (0-2h, 2-24h, and 24-48h) in postoperative period.

The overall PONV scores and postoperative nausea scores during 0-2 and 24-48 hours were comparable between the two groups. However, there was a significantly lower PONV score (p = 0.023) and postoperative nausea score (p = 0.010) during 2-24 hours in group B (palonosetron) compared to group A (ondansetron). During 2-24 h postoperative period, 63% were free from PONV in group B compared to 40% in group A (Figure 2), and 66% of patients in group B were free from nausea compared to only 40% in group A (p <0.05) (Tables 3-4).

**Table 3:** Postoperative nausea and vomiting (PONV) score. Values are number of patients (%); *p < 0.05 for group B compared with group A.

Overall PONV score		Group A (n = 65)	Group B (n = 65)	P-value
		Frequency (%)	Frequency (%)	
0-2h	0	51 (78)	48 (74)	0.218
	1	7 (11)	14 (21)	
	2	2 (3)	0 (0)	
	3	5 (8)	3 (5)	
2-24 h	0	26 (40)	41 (63)	0.023*
	1	22 (34)	16 (25)	
	2	0 (0)	0 (0)	
	3	17 (26)	8 (12)	
24-48 h	0	58 (89)	57 (88)	0.177
	1	6 (9)	8 (12)	
	2	0 (0)	0 (0)	
	3	1 (2)	0 (0)	

**Table 4:** Postoperative nausea score. Values are number of patients (%); *p < 0.05 for group B compared with group A.

Overall nausea score		Group A (n = 65)	Group B (n = 65)	P-value
		Frequency (%)	Frequency (%)	
0-2 h	0	51 (78.4)	50 (77)	0.101
	1	7 (10.8)	13 (20)	
	2	0 (0)	0 (0)	
	3	7 (10.8)	2 (3)	
2-24 h	0	26 (40)	43 (66)	0.010*
	1	24 (37)	15 (23)	
	2	0 (0)	0 (0)	
	3	15(23)	7 (11)	
24-48 h	0	58 (89)	56 (86)	0.593
	1	7 (11)	9 (14)	
	2	0 (0)	0 (0)	
	3	0 (0)	0 (0)	

Postoperative vomiting score or retching during 0-2 h, 2-24 h, and 24-48 h between the two groups were comparable (Table 5). 

**Table 5:** Postoperative vomiting score. Values are the number of patients (%).

Overall vomiting score		Group A (n = 65)	Group B (n = 65)	P-value
		Frequency (%)	Frequency (%)	
0-2 h	0	59 (91)	61 (95)	0.061
	1	6 (9)	1 (2)	
	2	0 (0)	0 (0)	
	3	0 (0)	2 (3)	
2-24 h	0	48 (74)	56 (86)	0.173
	1	11 (17)	7 (11)	
	2	0 (0)	0 (0)	
	3	6 (9)	2 (3)	
24-48 h	0	64 (98.5)	65 (100)	1.000
	1	1 (1.5)	0 (0)	
	2	0 (0)	0 (0)	
	3	0 (0)	0 (0)	

**Figure 3:**
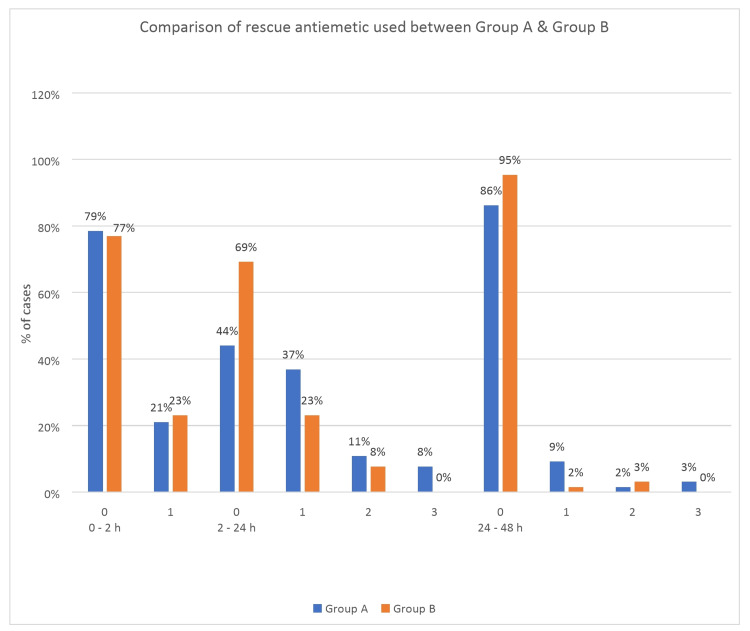
Comparison of the amount of ondansetron (first-line rescue antiemetic drug) used between Group A (ondansetron) and Group B (palonosetron). 0,1,2,3 on the x-axis denotes the number of times rescue antiemetic drugs were administered during a time interval (0-2h, 2-24h, and 24-48h) in the postoperative period.

The amount of first-line rescue antiemetic (ondansetron) used during 2-24 hours was significantly higher in group A than in group B (p = 0.012), whereas the amount of dexamethasone (second-line or ultimate rescue antiemetic) used was similar in both the groups (Figure 3 and Table 6).

**Table 6:** Amount of ondansetron (first-line rescue antiemetic drug) used. Values are the number of patients (%).

	Amount of ondansetron (No. of doses)	Group A (n = 65)	Group B (n = 65)	P-value
		Frequency (%)	Frequency (%)	
0-2 h	0	51 (79)	50 (77)	0.833
	1	14 (21)	15 (23)	
2-24 h	0	29 (44)	45 (69)	0.012
	1	24 (37)	15 (23)	
	2	7 (11)	5 (8)	
	3	5 (8)	0 (0)	
24-48 h	0	56 (86)	62 (95)	0.102
	1	6 (9)	1 (2)	
	2	1 (2)	2 (3)	
	3	2 (3)	0 (0)	

A complete response to the drug in either group was comparable during 0-2h and 24-48 h, whereas during 2-24 h, complete response with palonosetron was significantly higher compared to ondansetron (63% vs. 40% for PONV and 69% vs. 44% for rescue antiemetic used, respectively; (p <0.05) (Tables 3 and Table 6) (Figures 2-3). The incidence of adverse effects was similar between the two groups (Table 7).

**Table 7:** Incidence of adverse events.

Adverse effects	Group A	Group B	P-value
	Frequency (%)	Frequency (%)	
Headache	10 (15)	8 (12)	0.800
Dizziness	4 (6)	8 (12)	0.364
Drowsiness	14 (22)	17 (26)	0.537
Constipation	4 (6)	14 (22)	0.020
Allergic Reaction	0 (0)	0 (0)	-
ECG Changes	0 (0)	0 (0)	-

Regarding patient satisfaction score, higher satisfaction was observed with palonosetron than ondansetron (89% vs. 77%), 3% in each group were dissatisfied, and the rest were neutral. However, this observation was not statistically significant.

## Discussion

PONV is observed after general, regional, and local anesthesia in a substantial proportion of patients even though antiemetic prophylaxis is used widely in modern anesthesia practice. The etiology/pathophysiology of PONV is multifactorial and includes patient-related factors (young age, female sex, anxiety, history of PONV/motion sickness, genetic predisposition gastroparesis), surgical factors (laparoscopy, middle ear surgery), and mode of anesthesia (total intravenous anesthesia [TIVA] or inhalational) [13,17,18]. The simplified scoring system by Apfel CC et al. established four predisposing factors which increase the probability of PONV by 18-22% per risk factor [2]. These factors were well adjusted in our study, and more than three risk factors as per this scoring system were present in both groups. Other factors like laparoscopic surgery (40-70% reported incidence) [3], surgery duration, and use of volatile anesthetics also contributed to PONV [13], increasing the risk for developing PONV. Thus, it was not ethically feasible to include a control/placebo group, and preventing PONV was prioritized similarly to treating postoperative pain.

Several limitations of studies in the literature include quality and design, variable inclusion criteria, non-uniform dosages, and measurement times, leading to clinical heterogeneity among the studies. One such meta-analysis has mentioned that more high-quality randomized controlled trials are needed to impart superior clinical evidence for rational clinical decisions regarding precise and effective choices for PONV prophylaxis in patients undergoing laparoscopic surgery [11]. However, parameters like patient demographics, anesthesia regimen, postoperative analgesics, duration, and type of surgery were comparable and well-controlled in our study. Hence, any variation in response is attributable to the characteristics and effects of study drugs.

Stimulation of 5-HT3 receptors is the main event involved in vomiting reflex initiation. Multifactorial agents and inputs arising from diverse areas are involved, which initiate this reflex centrally by stimulating the 5-HT3 receptors located on the chemoreceptive trigger zone (CTZ) in the medulla. Also, serotonin is released from small intestinal enterochromaffin cells, stimulating 5HT3 receptors on vagal afferent fibers [15]. The 5-HT3RA are used commonly as they are more efficacious in the treatment and prevention of PONV compared to other antiemetics and have an enviable safety profile with most side effects being mild and transient [4,5]. Palonosetron, a potent 5-HT3RA, has unique pharmacology, structure, and clinical effects with a longer half-life and a stronger affinity for receptor binding than older 5-HT3RA. Based on receptor binding studies, palonosetron interacts with 5-HT3 receptors in a manner different from ondansetron and granisetron by binding in an allosteric, positively cooperative manner at different sites [19]. In addition, it blocks substance-P-associated response, has negative cooperativity with neurokinin-1 receptors by crosstalk, and prolonged effects with regard to receptor-ligand binding and responsiveness to serotonin [20]. In adults, the elimination half-life of palonosetron is 40 h and may extend up to 48 h, in contrast to 3-6 h for ondansetron.

The consequences of PONV can vary from transient discomfort to serious complications, thus limiting the benefit of laparoscopy by delaying discharge or prolonging recovery. However, there is an increasing trend towards early discharge/enhanced recovery protocol (ERP) after surgery [13,21]. Therefore, a more potent and longer-acting drug will benefit such patients.

We undertook this study to gather more data to evaluate and compare the efficacy of palonosetron with ondansetron for PONV prophylaxis for 48 hours in high-risk patients undergoing laparoscopic gynecological surgery. Our study showed significantly lower overall PONV and nausea scores in the palonosetron group compared to the ondansetron group during 2-24 h (Tables 3-4; Figure 2). This could be explained by its better potency, longer half-life, and greater 5HT3 receptor affinity [19,20]. The comparable PONV and nausea score observed during 24-48 h may be explained by lesser exposure to risk factors during this period (washout of inhalational agents, metabolism of opioids used in PACU, no surgical stimuli, and use of non-emetogenic drugs for pain control). Park SK and Cho EJ compared ondansetron with palonosetron in laparoscopic gynecological surgery. They reported that incidence of PONV and nausea (not vomiting) was significantly lower with palonosetron compared to ondansetron during 0-24 h, which agrees with our study [16]. Similarly, Moon YE et al. studied PONV following thyroidectomies during the postoperative period (up to 24 h) and reported a higher incidence of PONV with ondansetron (62%) than palonosetron (42%) [22].

In our study, the frequency of vomiting in the ondansetron group (26%) was greater than in the palonosetron group (14%) during 2-24 h follow-up. However, this was not statistically significant, and the same holds true for 0-2 h and 24-48 h follow-up period (Table 5). Kazemi-Kjellberg F et al. [23] suggested that 5-HT3RA are very efficacious in controlling vomiting rather than nausea, which corroborates our finding. Also, perhaps due to multifactorial pathophysiology of PONV and involvement of several receptors in vomiting reflex (including serotonin 5-HT3, histamine H2, dopamine D2, alpha2 adrenergic, GABA, muscarinic cholinergic, and neurokinin1) [24], the difference in frequency of vomiting between groups could not attain statistical significance. More patients had retching in 2-24 h period in the ondansetron group (8% vs. none with palonosetron) but this was not statistically significant.

During the 2-24h period, patients who showed complete response were significantly greater with palonosetron than with ondansetron (Tables 3,6; Figures 2,3). A previous study by Park SK and Cho EJ [16] also reported similar findings, with more patients in the palonosetron group having a complete response than the ondansetron group. This finding also explains why the amount of first-line rescue antiemetic used was significantly higher in the ondansetron group than the palonosetron group during the 2-24 h period (56% vs. 31%) (Table 6; Figure 3). However, the amount of dexamethasone (second-line rescue antiemetic drug) required was comparable in both groups.

The timing of ondansetron administration has been a topic of debate. Although the drug manufacturers recommend administration before induction, the relatively short half-life (3.5-6 h) may decrease the antiemetic activity of ondansetron in procedures lasting over three hours. However, Joslyn AF et al. [25] in their study mentioned that the rationale behind the administration of ondansetron prior to the induction of anesthesia was that a more accurate assessment of adverse events could be done (injection site reactions, dizziness, or lightheadedness and changes in hemodynamic parameters, ECG changes). Also, we believe it is more pertinent in high-risk patients that a prophylactic drug is administered prior to induction to antagonize the proposed mechanism of PONV, rather than at the end of surgery when the receptor pathway would have been already stimulated.

In our study, more patients in the palonosetron group were satisfied (89%) compared to the ondansetron group (77%). This was not statistically significant but probably reflects the better antiemetic profile of palonosetron. The incidence of adverse effects was similar in both study groups suggesting a similar safety profile. Navari RM [26] found no clinically relevant differences among palonosetron, ondansetron, or dolasetron in the laboratory, electrocardiographic, or vital sign changes, which agree with our study. The findings by Park SK and Cho EJ corroborated our findings related to patient satisfaction scores and incidence of adverse effects [16]. We did not find any ECG changes after drug administration which correlates with Kim HJ et al., who studied the effect of palonosetron on QTc interval in patients undergoing sevoflurane anesthesia [27].

Consensus is emerging that antiemetic prophylaxis is not cost-effective in low-risk patients (10% or 20% expected risk) and is best accomplished in moderate, high-risk, or extremely high-risk patients with drug combinations. Single-dose palonosetron (longer half-life and better potency) seems more rational than multiple dosing with ondansetron, which might not be very desirable. Also, single dosing in operation theatre can reduce the chance of drug interaction later in the postoperative period and can mitigate the higher cost of the newly developed drug. The decision/commitment to treat patients depends on drug efficacy, baseline risk factors for PONV, adverse-effect profile, and cost of acquiring the drug, which are non-identical among different settings [5,13].

In the late recovery period, the sustained anti-nausea effect of palonosetron compared to ondansetron assumes notable significance in ambulatory/daycare and ERP settings. To date, palonosetron is proven to prevent PONV till 24 h of the postoperative period, and efficacy beyond 24 h has not yet been demonstrated [16,22]. Therefore, we extended the follow-up period up to 48h so we can suggest a cost-effective drug with cover extending to the post-discharge period leading to smoother recovery and probably decreased chances of readmission. In patients with a medium-to-high risk for developing PONV, combination therapy or multimodal approach with reliance on risk reduction strategy can better address this issue [13]. Also, recent guidelines reiterate and recommend multimodal prophylaxis when more than one risk factors are present [13].

There are some limitations to our study. First, even after following stringent exclusion criteria, we could not exclude medications for comorbidities like hypertension or diabetes mellitus that may influence the risk for PONV. Also, the postoperative antibiotic regimens may differ in patients and can account for differences in PONV incidence. Second, we could not evaluate the baseline incidence of PONV by including the placebo/control group as it would have been unethical to withhold prophylaxis for patients at high risk for PONV. Third, we did our study based on optimal doses of ondansetron and palonosetron without the knowledge of equipotent doses, and further studies are warranted to evaluate the equipotency of these drugs. Fourth, the administration time of ondansetron has been under debate for a long time. Manufacturers recommend administration before induction but considering its short half-life, it is debatable that the difference in complete response in the late postoperative period may not be seen had it been given towards the end of surgery. Fifth, subjectivity in assessing patient satisfaction is unavoidable to some degree. Sixth, there can be a concern regarding the use of ondansetron as rescue antiemetic in our study, but we wanted to avoid the use of butyrophenones, antihistaminics et al. as rescue antiemetic due to associated side effects and onset of action of dexamethasone is long, so we chose ondansetron due to the faster onset of action and ondansetron being part of a standard protocol for the management of PONV in our setup. Lastly, we do acknowledge that the recent trend is towards non-opioid-based anesthesia and the use of intraoperative opioids is a risk factor for PONV. To address and minimize the use of opioids in our study, we used a multimodal strategy for postoperative pain management, mainly with paracetamol and diclofenac. Morphine was used on an SOS basis only in PACU and in patients where the pain was not controlled with other strategies. It is standard practice in our setup and would be unethical to deny opioids to a patient experiencing severe pain. We standardized the use of intraoperative opioids in both groups, and there was no difference in postoperative morphine requirement between both groups. These limitations need to be addressed, and further multicenter studies with a large sample size may help provide data to overcome these shortcomings.

## Conclusions

Our study demonstrated that palonosetron produced a significantly lower incidence of overall PONV and postoperative nausea scores during a 2-24h period compared with ondansetron in high-risk patients undergoing laparoscopic gynecological surgery. The comparable PONV characteristics in study groups in the postoperative phase (0-2 h, 24-48 h) with a significant difference in response during 2-24 h (better anti-nausea effect, decrease in overall incidence of PONV, lesser need for rescue antiemetics postoperatively) emphasize higher efficacy and potency of palonosetron in long-term prophylaxis. This also merits the use of palonosetron in ambulatory/daycare surgery and ERP settings and surgeries associated with high-risk PONV, thus ensuring smooth recovery and recuperation. In addition, a single-dose regimen of palonosetron can decrease the requirement for multiple administrations postoperatively as needed with ondansetron. Thus this might prove to be cost-beneficial in the long term.

## Figures and Tables

**Figure 4:**
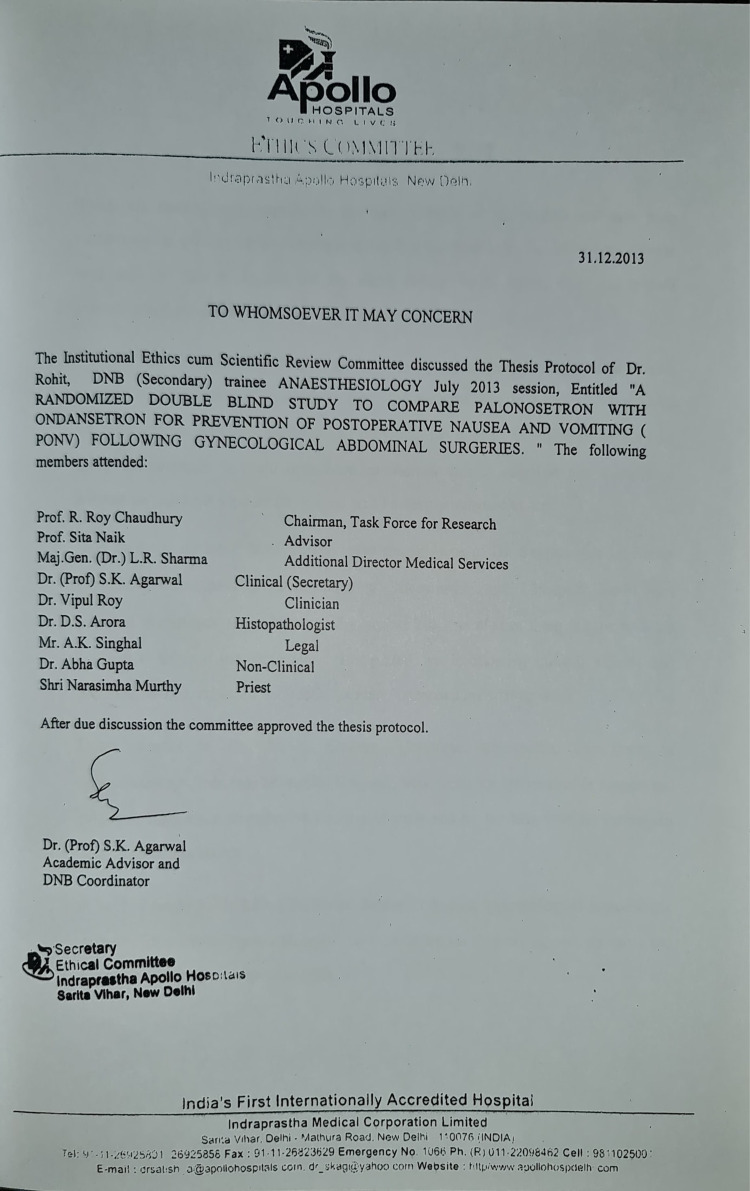
Ethics committee approval.
